# Transient receptor potential melastatin 2 channels are overexpressed in myalgic encephalomyelitis/chronic fatigue syndrome patients

**DOI:** 10.1186/s12967-019-02155-4

**Published:** 2019-12-03

**Authors:** Cassandra Balinas, Helene Cabanas, Donald Staines, Sonya Marshall-Gradisnik

**Affiliations:** 1 The National Centre for Neuroimmunology and Emerging Diseases, Menzies Health Institute Queensland, Griffith University, Southport, Gold Coast, QLD 4222 Australia; 2 Consortium Health International for Myalgic Encephalomyelitis, National Centre for Neuroimmunology and Emerging Diseases, Griffith University, Gold Coast, QLD Australia

**Keywords:** Adenosine diphosphate ribose, Calcium, Myalgic encephalomyelitis/chronic fatigue syndrome, Interleukin-2, Natural killer cells, Transient receptor potential melastatin 2

## Abstract

**Background:**

Myalgic encephalomyelitis/chronic fatigue syndrome (ME/CFS) is hallmarked by a significant reduction in natural killer (NK) cell cytotoxicity, a mechanism tightly regulated by calcium (Ca^2+^). Interestingly, interleukin-2 (IL-2) increases NK cell cytotoxicity. Transient receptor potential melastatin 2 (TRPM2) ion channels are fundamental for Ca^2+^ signalling in NK cells. This pilot investigation aimed to characterise TRPM2 and CD38 surface expression in vitro on NK cells in ME/CFS patients. This investigation furthermore examined the pharmaceutical effect of 8-bromoadenosine phosphoribose (8-Br-ADPR) and *N*_6_-Benzoyladenosine-3′,5′-cyclic monophosphate (*N*_6_-Bnz-cAMP) on TRPM2 and CD38 surface expression and NK cell cytotoxicity between ME/CFS and healthy control (HC) participants.

**Methods:**

Ten ME/CFS patients (43.45 ± 12.36) and 10 HCs (43 ± 12.27) were age and sex-matched. Isolated NK cells were labelled with fluorescent antibodies to determine baseline and drug-treated TRPM2 and CD38 surface expression on NK cell subsets. Following IL-2 stimulation, NK cell cytotoxicity was measured following 8-Br-ADPR and *N*_6_-Bnz-cAMP drug treatments by flow cytometry.

**Results:**

Baseline TRPM2 and CD38 surface expression was significantly higher on NK cell subsets in ME/CFS patients compared with HCs. Post IL-2 stimulation, TRPM2 and CD38 surface expression solely decreased on the CD56^Dim^CD16^+^ subset. 8-Br-ADPR treatment significantly reduced TRPM2 surface expression on the CD56^Bright^CD16^Dim/−^ subset within the ME/CFS group. Baseline cell cytotoxicity was significantly reduced in ME/CFS patients, however no changes were observed post drug treatment in either group.

**Conclusion:**

Overexpression of TRPM2 on NK cells may function as a compensatory mechanism to alert a dysregulation in Ca^2+^ homeostasis to enhance NK cell function in ME/CFS, such as NK cell cytotoxicity. As no improvement in NK cell cytotoxicity was observed within the ME/CFS group, an impairment in the TRPM2 ion channel may be present in ME/CFS patients, resulting in alterations in [Ca^2+^]_i_ mobilisation and influx, which is fundamental in driving NK cell cytotoxicity. Differential expression of TRPM2 between NK cell subtypes may provide evidence for their role in the pathomechanism involving NK cell cytotoxicity activity in ME/CFS.

## Background

Myalgic encephalomyelitis/chronic fatigue syndrome (ME/CFS) is a disabling condition characterized by unexplained chronic fatigue that is associated with immune, neurological (including autonomic), musculoskeletal, cardiovascular and gastrointestinal symptoms [1, 2]. Currently, accurate diagnosis remains challenging in the absence of a clinical or laboratory test. Although the aetiology of ME/CFS remains undefined, immunological dysfunction has been consistently implicated in this condition. Notably, a significant reduction in natural killer (NK) cell cytotoxicity is a consistent feature in ME/CFS patients compared with healthy control (HC) participants [3–6].

NK cells are granular lymphocytes of the innate immune system, principally responsible for recognising and responding to pathogen invasion [7]. NK cells are also essential to combat viral and microbial infection, as well as tumour development [7]. NK cells are located in peripheral blood, spleen, bone marrow and lymph nodes, and stem from the CD34 hematopoietic progenitor cell lineage [7]. At least five NK populations have been classified, however the two predominant human subsets include CD56^Dim^CD16^+^ and CD56^Bright^CD16^dim/−^ populations. Approximately 90% of peripheral NK cells are CD56^Dim^CD16^+^ which are highly cytotoxic and kill infected, tumour or ‘missing self’ cells [8]. Comparatively, the CD56^Bright^CD16^Dim/−^ subset are primarily involved in immunosurveillance and cytokine production [9]. Interestingly, IL-2-activated NK cells are more lytic to target cells than resting NK cells [10–12].

The IL-2 receptor (IL-2R) is a heterotrimeric protein expressed on a number of immune cells that binds and responds to IL-2 [13, 14]. The IL-2R comprises three forms: α (alpha) (also called IL-2Rα, CD25), β (beta) (also called IL-2Rβ or CD122) and γ (gamma) (also called IL-2Rγ or CD132). Binding of IL-2 promotes Janus tyrosine Kinase (JAK) 1 and JAK 3 enzyme activation, in turn initiating the Mitogen-Activated Protein Kinase (MAPK), phosphoinositide 3-kinase (PI_3_K), and the Signal Transducer and Activator of Transcription (STAT) pathways [15]. Activation of these pathways enhances NK cell cytotoxic function, known as lymphokine-activated killing (LAK). NK cells are the predominant effector cells within LAKs, which are mechanistically equivalent to peripheral blood NK cells [16], but possess enhanced cytotoxic potential against an extensive spectrum of cell targets [17]. IL-2 has been postulated to be possibly involved in the de novo expression of proteins that act between CD38 and the lytic machinery in NK cells [10].

Calcium (Ca^2+^) plays a fundamental role in various cellular mechanisms such as intracellular signalling pathways, cell differentiation, cell division, apoptosis and transcriptional events. Notably, intracellular Ca^2+^ is crucial for target cell adhesion, immune synapse formation, granule polarization and degranulation, all of which are essential for NK cells to mediate their natural cytotoxicity [8, 18]. One fundamental signalling pathway involved in intracellular Ca^2+^ is the store-operated calcium entry (SOCE) mechanism [19]. Recently, the transient receptor potential channel (TRP) family has emerged as a potential SOCE candidate [19, 20].

TRP channels are a unique group of ion channels that function as polymodal cell sensors due to their extensive sensitivity to physical and chemical stimuli. TRP channel activation follows deviations in the cellular environment, including pathogens, temperature, pressure, chemicals, oxidation and reduction, toxins, osmolarity and pH [21, 22]. Transient receptor potential melastatin 2 (TRPM2) is a homo-tetrameric nonselective cation permeable channel localised in the plasma membrane and lysosomal compartments [23]. The protein is highly expressed in the brain, immune system, endocrine cells, and endothelia [24]. TRPM2 uniquely possesses dual ion channel functionality and enzymatic ADP-ribose (ADPR) hydrolase activity [21].

TRPM2 is primarily activated in a synergistic fashion by low micromolar levels of cytosolic ADPR and Ca^2+^ [23]. At a cellular level, ADPR is predominantly produced by the hydrolysis of NAD^+^ and/or cADPR by glycohydrolases, including the ectoenzyme, CD38 [23]. CD38 uses NAD^+^ to generate various Ca^2+^ mobilizing secondary messengers, such as ADPR, cADPR and NAADP [23, 24]. Direct binding to the enzymatic NUDT9-H domain activates and opens the channel, subsequently facilitating the permeation of sodium (Na^2+^), potassium (K^+^) and Ca^2+^ into the cell and hydrolysis of ADPR to ribose 5-phosphate and adenosine monophosphate (AMP) [25]. Importantly, TRPM2 activation increases intracellular free calcium concentration ([Ca^2+^]_i_) [26, 27], which can mediate signalling roles in inflammatory and secretory by releasing vesicular mediators (i.e. cytokines, neurotransmitters), as well as apoptosis and necrotic cell death under oxidative stress [23].

A recent in vivo mouse investigation discovered a novel anti-tumour mechanism in NK cells via a ADPR-CD38 synergy with TRPM2 [28]. This investigation highlighted three fundamental steps of this synergistic pathway: (1) activation of intracellular CD38 by protein kinase A following NK cell recognition of a tumour cell results in ADPR production; (2) ADPR targets TRPM2 channels on cytolytic granules, and (3) TRPM2-mediated Ca^2+^ signalling causes cytolytic granule polarisation and degranulation, resulting in anti-tumour activity [28]. These results suggest that CD38, ADPR and TRPM2 are key components in mediating Ca^2+^-induced anti-tumour activity in NK cells [28].

Pharmacologically, TRPM2 currents can be inhibited by altering the production of TRPM2 secondary messengers [24]. Pharmacological antagonists reviewed in the literature include: 8-Br-cADPR (cADPR antagonist) [29], 8-Br-ADPR (ADPR antagonist) [30], nifedipine, econazole [31] flufenamic acid [31], imidazole antifungal agents [31], anthranilic acid, *N*-(*p*-amycinnamoyl) [32] and 2-APB [32]. However, majority of these molecules are insufficiently potent and do not exhibit high TRPM2 specificity [24].

To date, no potent pharmacological TRPM2 agonists have been reported, however *N*_6_-Benzoyladenosine-3′,5′-cyclic monophosphate (*N*_6_-Bnz-cAMP) has been identified as a weak TRPM2 agonist through indirect stimulation via protein kinase A (PKA) production, most likely through phosphorylation [33].

Characterisation of TRPM2 has predominantly been studied with in vivo models, primarily by genetic knockdown [28, 33–40]. Although in vitro investigations of TRPM2 have also been examined in T cells, macrophages, SCC9 cells, and HEK293 cells [36, 39, 41, 42] there have been no in vitro TRPM2 models studied on human NK cells. Given TRPM2 is critical for Ca^2+^ homeostasis and NK cell cytotoxicity, this is the first in vitro investigation to examine NK cell cytotoxicity following TRPM2 modulation with *N*_6_-Bnz-cAMP and 8-Br-ADPR in ME/CFS patients. Secondly, this study aims to characterise TRPM2 surface expression on NK cell subsets to determine whether differential expression of TRPM2 plays a role in ME/CFS patients compared with HC.

## Methods

### Study participants

A total of 11 ME/CFS and 11 HC participants were selected for this pilot investigation. Two participants were excluded due to outlier values. Participants were sourced from the National Centre of Neuroimmunology and Emerging Diseases (NCNED) database for ME/CFS between January and April 2019. Participants were excluded if they were pregnant or breastfeeding, or reported a previous history of smoking, alcohol abuse or chronic illness (for example, autoimmune diseases, cardiac diseases and primary psychological disorders). Participants donated 85 ml of whole blood in ethylenediaminetetraacetic acid (EDTA) tubes between 8:30 am and 10:00 am on the Gold Coast, Queensland, Australia. Full blood count was performed within 4 h (hr) of blood collection for each participant. All participants provided written consent and the study was approved by the Griffith University Human Research Ethics Committee (HREC/15/QGC/63).

### Peripheral blood mononuclear cell isolation and natural killer cell isolation

Peripheral blood mononuclear cells (PBMCs) were isolated from whole blood by centrifugation over a density gradient medium (Ficoll-Paque Premium; GE Healthcare, Uppsala, Sweden) to separate granulocytes as previously described [3, 43]. PBMCs were stained with trypan blue stain (Invitrogen Life Technologies, Carlsbad, CA, USA) to determine total cell count and cell viability and adjusted to a final concentration of 5 × 10^7^ cells/ml. NK cells were isolated from PBMCs using an EasySep Negative Human NK Cell Isolation Kit (Stemcell Technologies, Vancouver, BC, Canada). NK cell purity was measured following staining with CD56-Pe-Cy7 (0.25 µg/5 µl) and CD3-APC-H7 (0.5 µg/5 µl) antibodies (Beckon Dickinson [BD] Bioscience, Miami, FL, USA) for 20 min (min) at room temperature in the dark and analysed using a LSR-Fortessa X20 flow cytometer (BD Biosciences, Miami, FL, USA). NK cell purity was 92.46 ± 2.375 for HCs and 93.58 ± 0.9282 for ME/CFS, respectively (Additional file 1).

### Interleukin-2 stimulation

NK cells were stimulated with 20 IU/ml of recombinant human IL-2 (specific activity 5 × 10^6^ IU/mg) per test (Miltenyi Biotech, BG, Germany) and incubated for 24 h at 37 °C with 5% CO_2_ in Roswell Park Memorial Institute medium (RPMI)-1640 (Invitrogen Life Technologies, Carlsbad, CA, USA) supplemented with 10% fetal bovine serum (FBS) (Invitrogen Life Technologies, Carlsbad, CA, USA).

### Drug treatment

IL-2 stimulated NK cells were treated with the following drugs at a final concentration of 100 μM *N*_6_-Bnz-cAMP, and 100 μM 8-Br-ADPR for 30 min at 37 °C with 5% CO2 in RPMI-1640 (Invitrogen Life Technologies, Carlsbad, CA, USA) supplemented with 10% FBS (Invitrogen Life Technologies, Carlsbad, CA, USA)). All drugs were purchased from Bio-Techne (Tocris Bioscience, Sussex, UK). Labelled cells were washed with 2 ml of RPMI-1640 supplemented with 10% FBS and centrifuged at 250 g for 5 min. Supernatant was removed prior to NK lysis and TRPM2 immunophenotyping.

### TRPM2 immunophenotyping assay

Following magnetic NK cell isolation, baseline TRPM2 and CD38 surface expression was measured as previously described [44] NK cells were stained with trypan blue stain (Invitrogen Life Technologies, Carlsbad, CA) to determine live cell count and cell viability and adjusted to a final concentration of 1.1 × 10^6^ cells/ml. NK cells were incubated with an Fc receptor Blocking reagent (Miltenyi Biotech, Bergisch Gladbach. Germany) for 10 min at 4 °C prior to antibody staining. NK cells were incubated with primary fluorochrome labelled antibodies [CD3-APCH7 (0.5 µg/5 µl), CD56-PeCy7 (0.25 µg/5 µl), CD16-BV650 (0.25 µg/5 µl), and CD38-BV480 (1 µg/5 µl)] purchased from BD Biosciences), in addition to an unconjugated rabbit IgG polyclonal extracellular TRPM2 antibody (1:50) (OST00112W) (Thermo Fisher Scientific, Waltham, MA, USA) for 2 h at 4 °C in the dark.

Labelled cells were washed with stain buffer (BD Biosciences, Miami, FL, USA) and centrifuged at 350*g* for 5 min. Supernatant was removed and cells were incubated with a secondary Goat F(ab) Anti-Rabbit IgG H&L Fluorescein isothiocyanate (FITC) (1:500) (ab7050) (Abcam, UK) in 200 μl for 1 h at 4 °C in the dark. Cells were washed and stained with 5 µl of 7-AAD (BD Bioscience, New Jersey, USA) to measure cell viability. Cells were resuspended in 200 µl of stain buffer (BD Bioscience, Miami, FL, USA) and acquired at 10,000 events using the LSRFortessa X-20. Furthermore, TRPM2 and CD38 surface expression was measured following drug treatment.

Normal rabbit serum (1:50) (01-6101) (Thermo Fisher Scientific, Waltham, MA, USA) was used as a negative control to determine an individualised positive TRPM2 gate for each participant (Additional file 2). Additionally, an unstained tube (unlabelled NK cells); a secondary tube (secondary antibody only); and a Fluorescence Minus One (FMO) (CD56, CD3, CD16 and CD38) control were performed for each participant. Normalised TRPM2 and CD38 surface expression was calculated by compensating the percentage of fluorescence spill over into the B525/50 (TRPM2) and V525/50 (CD38) as outlined below for TRPM2:

$$TRPM2\,Surface\,Expression\, = \,TRPM2\,Stained\,tube\,\left( {parent \, \% } \right)\, - \,Normal\,Rabbit\,Serum\,\left( {parent \, \% } \right)$$

### Natural killer cell cytotoxicity assay

NK cytotoxic activity was conducted as previously described [43, 45, 46]. NK cells were labelled with Paul Karl Horan (PKH-26) (3.5 µl/test) Sigma-Aldrich, St. Louis. MO, USA) for 5 min (and incubated with K562 cells for 4 h at 37 °C with 5% CO_2_ in RPMI-1640 supplemented with 10% FBS. The concentration of NK cells and K562 cells was adjusted to 5 × 10^5^ cells/ml and 1 × 10^6^ cells/ml, respectively. During incubation, cells were combined at effector target (E:T) ratios of 25:1, 12.5:1 and 6.25:1 in addition to control samples (only 25:1 NK lysis ratio shown). Control and IL-2 treated cells were stained using Annexin V (2.5 µl/test) (BD Bioscience, Miami, FL, USA) and 7-AAD (2.5 µl/test) (BD Bioscience, Miami, FL, USA) to determine apoptosis using flow cytometry analysis recording 10,000 events. E:T ratio of 25:1, 12.5:1, 6.25:1 (only 25:1 NK lysis ratio shown) and control were used to assess cytotoxic activity. NK cytotoxic activity was calculated as percent specific death of the K562 cells for the three E:T ratios as previously described [43, 45, 46] and outlined below:

$$Cytotoxicity\,\left( \% \right) = \frac{{\left( {early\;stage\;apoptosis\; + \;late\;stage\;apoptosis\; + \;dead\;K562\;cells} \right)}}{All\;K562\;Cells}\; \times \;100$$

### LSR Fortessa X-20 flow cytometry analysis

Lymphocyte populations were identified using forward and side scatter dot plots. Exclusions were CD3^+^ cells and only CD3^−^ lymphocytes were further used to characterise NK cell subset populations using CD56 and CD16. NK cell subsets were characterised using the surface expression of CD56^Bright^CD16^Dim/−^NK cells and CD56^Dim^CD16^Bright/+^ NK cells. TRPM2 and CD38 surface expression was measured as percentage of parent cells (%) and NK cytotoxicity was measured as percentage of K562 cell death (%).

### Statistical analysis

Pilot data from this investigation were analysed using SPSS version 24 (IBM Corp, Version 24, Armonk, NY, USA) and GraphPad Prism, version 7 (GraphPad Software Inc., Version 7, La Jolla, CA, USA). Shapiro–Wilk normality tests were conducted to determine the distribution of data, in addition to skewness and kurtosis tests to determine data normality. The independent Mann–Whitney U test was performed to determine the statistical significance between and within groups in TRPM2 parameters on NK cells. Significance was set at p < 0.05 and the data are presented as mean ± standard error of the mean unless otherwise stated.

## Results

### Participant demographics

A total of 20 participants (10 HC and 10 ME/CFS patients) were included for this present study and no significant differences in age and gender were reported between groups (Table 1). Additionally, ME/CFS patients reported greater impairment compared with HCs in all Short-Form Health Survey (SF-36) subscales (lower score indicate greater impairment; p < 0.001), with the exception of emotional wellbeing. Full blood count parameters were measured for each participant. All participant results were within the specified reference ranges for each parameter and there were no significant differences between groups for these reporting parameters (Table 1).

**Table 1:** Participants’ demographic and serology results

Category	Item	HC	ME/CFS	p value
General demographics	Age (years)	43.10 ± 4.089	43.50 ± 4.126	0.989
	Gender			> 0.999
	Male (%, n)	(40%, 4)	(40%, 4)	
	Female (%, n)	(60%, 6)	(60%, 6)	
	BMI (kg/m^2^)	23.41 ± 0.881	25.21 ± 0.937	0.120
WHODAS		1.382 ± 0.518	38.73 ± 5.504	*< 0.0001*
Illness demographics (SF-36)	Pain (%)	85.91 ± 5.026	28.18 ± 7.971	*< 0.0001*
	Physical Functioning (%)	98.64 ± 0.975	35.45 ± 6.085	*< 0.0001*
	Role physical (%)	97.15 ± 1.547	13.08 ± 5.686	*< 0.0001*
	General Health (%)	74.99 ± 4.171	30.67 ± 3.988	*< 0.0001*
	Social functioning (%)	95.45 ± 3.049	28.41 ± 7.922	*< 0.0001*
	Emotional wellbeing (%)	94.45 ± 3.049	74.02 ± 9.277	0.069
Serology report	WBC	5.945 ± 0.371	6.909 ± 0.539	0.223
	Lymphocytes	1.705 ± 0.127	1.947 ± 0.106	0.223
	Neutrophils	3.575 ± 0.288	4.241 ± 0.501	0.519
	Monocytes	0.466 ± 0.051	0.502 ± 0.051	0.467
	Eosinophils	0.150 ± 0.031	0.518 ± 0.051	0.935
	Basophils	0.046 ± 0.005	0.048 ± 0.006	0.955
	Platelets	252.8 ± 24.12	246.8 ± 9.213	0.797
	RBC	4.665 ± 0.102	4.808 ± 0.165	0.835
	Haematocrit	0.416 ± 0.009	0.428 ± 0.013	0.618
	Haemoglobin	136.2 ± 3.261	142.9 ± 4.089	0.339

Data are represented as mean ± SEM using Mann Whitney U tests

*HC* healthy controls, *ME/CFS* myalgic encephalomyelitis/chronic fatigue syndrome, *BMI* body mass index, *RBC* red blood cell, *SF-36* short-form health survey, *WBC* white blood cell, *WHODAS* world health organisation disability assessment schedule

*** p < 0.0001

## Discussion

We have previously determined an optimal in vitro methodology to phenotype TRPM2 and CD38 surface expression on human NK cell subsets from HC participants using flow cytometry [44]. This current investigation is the first in vitro study to characterise TRPM2 and CD38 surface expression on peripheral NK cell subsets from ME/CFS patients. This is also the first study to examine the pharmacological effect of 8-Br-ADPR and *N*_6_-Bnz-cAMP drug treatments on TRPM2 and CD38 surface expression, as well as NK cell cytotoxicity in ME/CFS patients.

**Fig. 1:**
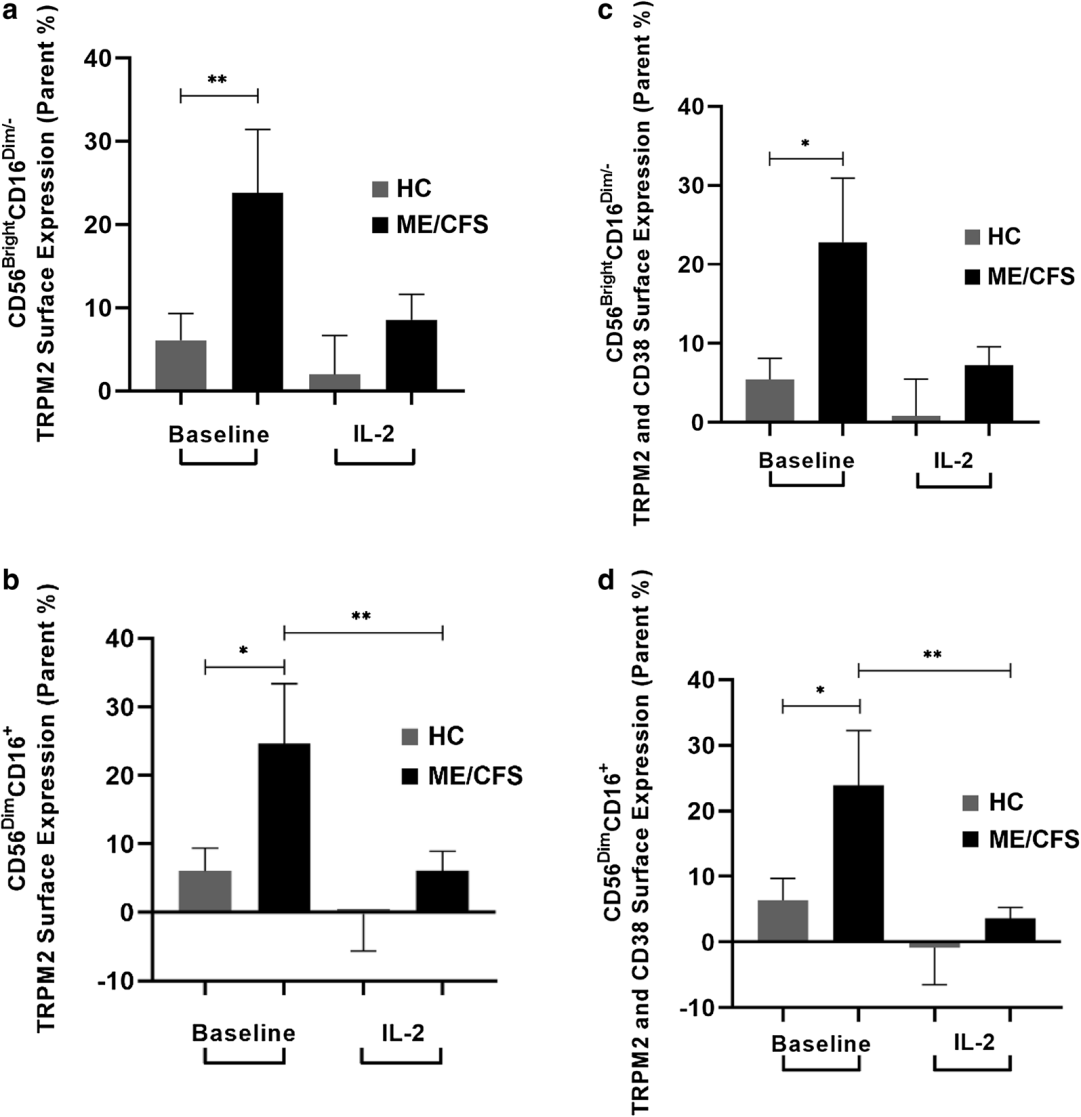
TRPM2 and CD38 surface expression on CD56^Bright^CD16^Dim/−^ and CD56^Dim^CD16^+^ NK cell subsets between groups post IL-2 stimulation. At baseline, TRPM2 surface expression was significantly higher in the ME/CFS group compared to HCs on CD56^Bright^CD16^Dim/−^ (**a**) and CD56^Dim^CD16^+^ NK cells (**b**). A consistent finding was found at dual expression with CD38 on both NK cell subsets (**c**, **d**). Post IL-2 stimulation, TRPM2 with and without CD38 significantly decreased on the CD56^Dim^CD16^+^ subset within the ME/CFS group (**b**, **d**). No significant differences in TRPM2 and CD38 surface expression were found within the HC group pre and post IL-2 stimulation in either NK cell subset

**Fig. 2:**
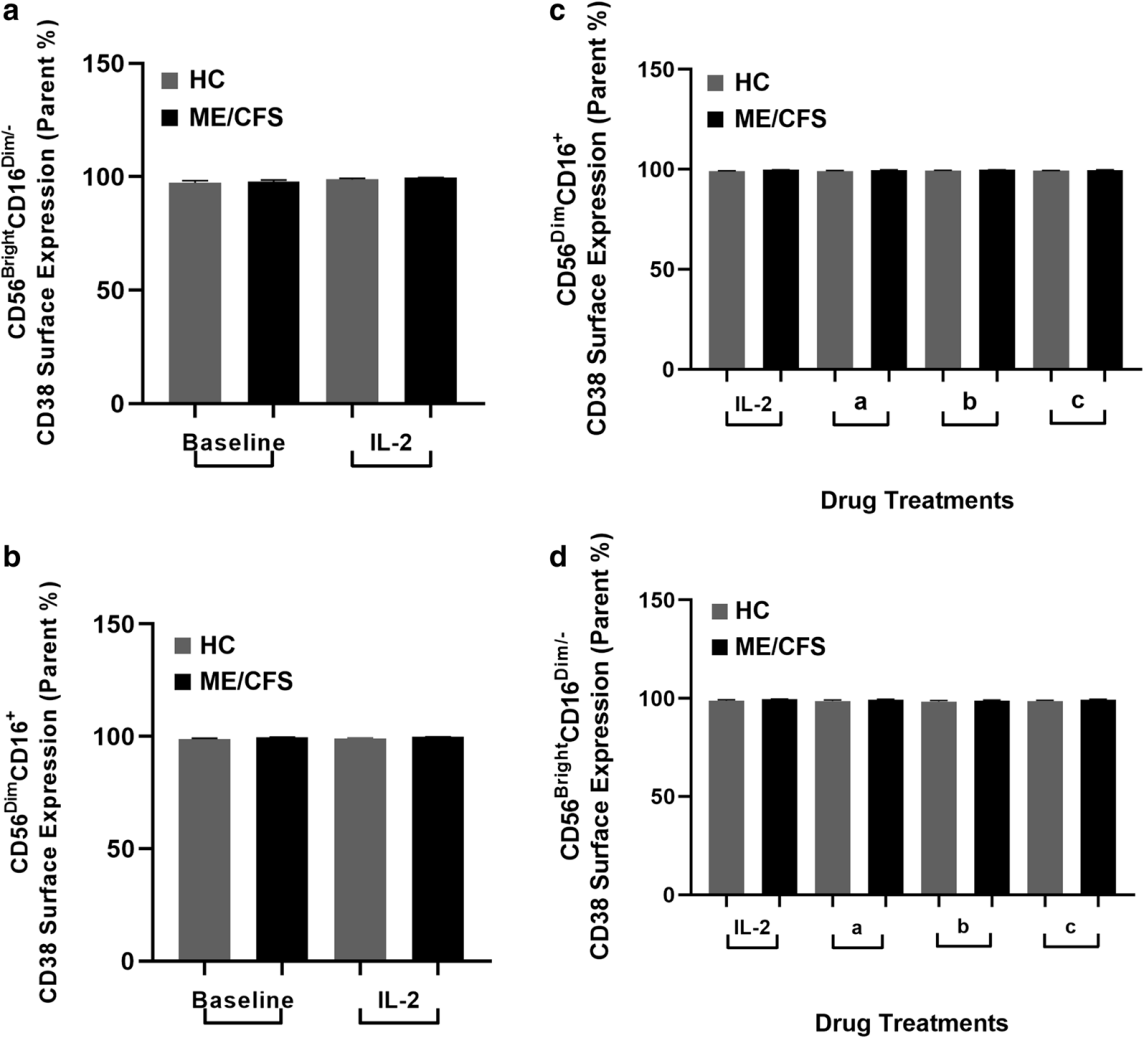
CD38 surface expression on CD56^Bright^CD16^Dim/−^ and CD56^Dim^CD16^+^ NK cell subsets between groups post IL-2 stimulation. No significant difference in CD38 surface expression was found between groups or within either NK cell subset pre and post IL-2 stimulation (**a**, **b**). No significant changes in CD38 surface expression was observed post drug treatment between or within groups on either NK cell subset (**c**, **d**)

At baseline, TRPM2 surface expression was significantly higher in ME/CFS patients compared with HCs on CD56^Bright^CD16^Dim/−^ and (Fig. 1a) and CD56^Dim^CD16^+^ NK cells (Fig. 1b). These findings were also found at dual expression with CD38 on both NK cell subsets (Fig. 1c, d). CD38 surface expression alone was reportedly higher in ME/CFS and HC participants (99%) on both NK cell subsets (Fig. 2a, b). However, when compared with dual expression with TRPM2, CD38 surface expression decreased to 22% (ME/CFS) and 6% (HC) on both subsets (Fig. 1c, d). This difference with co-expression is reflective of CD38’s additional functions, independent of TRPM2, such as cell adhesion, signal transduction and Ca^2+^ signalling. However, as CD38 surface expression did not differ between groups, our results highlight an overexpression of the TRPM2 ion channel within the ME/CFS group. In comparison to the reductions in TRPM3 surface expression reported in our previous findings [45, 47], we postulate that this overexpression in TRPM2 may function as a compensatory mechanism to alert a dysregulation in Ca^2+^ homeostasis within the NK cell.

Ca^2+^ plays a fundamental role in intracellular signalling pathways, cell differentiation and cell division, apoptosis and transcriptional events [22–24]. Upon stimulation, Ca^2+^ permeable TRP channels, such as TRPM2, generate changes in [Ca^2+^]_i_, by acting as Ca^2+^ gatekeepers via the plasma membrane. Notably, [Ca^2+^]_i_ is important to sensitise TRPM2 for activation by ADPR resulting in a positive feedback loop and Ca^2+^ entry [48, 49]. Changes in channel stoichiometry and assembly can induce significant dysregulations in Ca^2+^ mobilization [50, 51]. Thus, Ca^2+^ homeostasis requires a tight and meticulous regulation for efficient receptor functionality. An important Ca^2+^-dependent mechanism regulated by TRPM2 in NK cells is cytotoxic function.

**Fig. 3:**
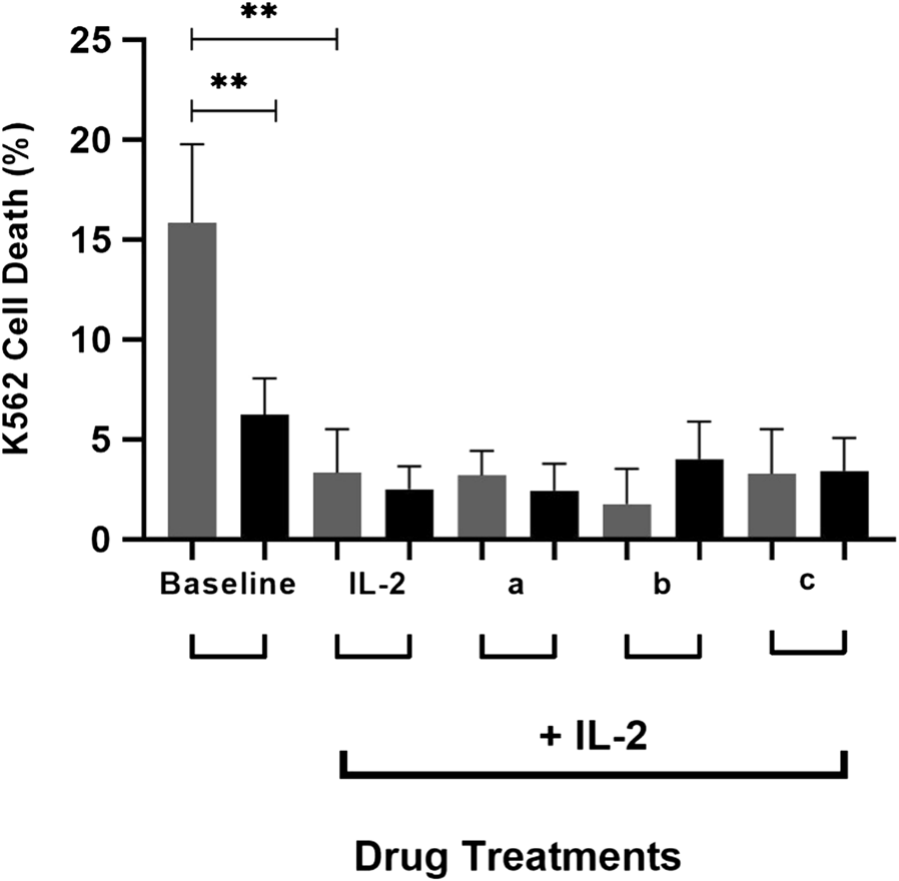
Natural killer cell cytotoxicity after treatment with 8-Br-ADPR and *N*_6_-Bnz-cAMP between groups. At baseline, a significant increase in NK cell cytotoxicity was observed in HC participants compared with ME/CFS patients. Within the HC group, NK cell lysis decreased post IL-2 stimulation. This observation was not found within the ME/CFS group. No significant difference in NK cell cytotoxicity was observed between groups after drug treatments

Although the underlying aetiology of ME/CFS remains unknown; a significant reduction in NK cell cytotoxicity is a consistent laboratory finding in ME/CFS patients compared with HCs [3–6], which was confirmed in this present study (Fig. 3). In the ME/CFS group, an improvement in NK cell function was expected in correlation to the overexpression of TRPM2 as a compensatory mechanism (Fig. 1). However, as baseline NKcell cytotoxicity was significantly reduced in ME/CFS patients compared with HCs, these results may suggest an impaired and/or faulty TRPM2 ion channel within the ME/CFS group. An impairment in the TRPM2 ion channel function may prevent the permeabilization and influx of Ca^2+^ within the NK cell; resulting in a subsequent reduction in Ca^2+^ modulation and [Ca^2+^]_i_, thus leading to impaired Ca^2+^-dependent mechanisms, including NK cell cytotoxicity.

Following IL-2 stimulation, NK cell cytotoxicity significantly decreased within the HC group (Fig. 3). This was an unexpected outcome as previous investigations have reported enhancements in NK cell cytotoxicity following IL-2 stimulation [10–12]. However, an in vivo mouse study discovered that IL-2 rapidly lowers the activation threshold of NK cells to adhere and engage with their targets [12]. Moreover, NK cell responses did not augment post IL-2 in the presence of inhibitory receptor signalling, suggesting potential interactions of the IL-2R with integrin or activating-receptor signalling pathways [12]. An additional rationale could reflect the limited culture time period (24 h), as most NK cell cultures range between 1 and 2 weeks [52–56]. However, as fresh human peripheral NK cells were used, a longer culture time period was not preferential as NK cell purity significantly decreases to 40–50% [57]. Conversely, no change in NK cell cytotoxicity was observed post IL-2 stimulation within the ME/CFS group (Fig. 3), which reinforces a significant impairment in the TRPM2 ion channel.

Interestingly, TRPM2 and CD38 surface expression significantly decreased after IL-2 stimulation in ME/CFS patients (Fig. 1). However, this finding was only observed on the CD56^Dim^CD16^+^ NK cell subset within the ME/CFS group (Fig. 1a, c). A possible rationale may involve downregulation the IL-2Rα on the CD56^Dim^CD16^+^ subset, which has been suggested to reduce the ability for efficient CD56^Dim^CD16^+^ NK cell activation and restoration of proliferative capability in response to IL-2 [10].

Thus, a negative feedback mechanism, involving Ca^2+^, may be present between the IL-2Rα and TRPM2 and CD38 on CD56^Dim^CD16^+^ NK cells in response to IL-2 as it has been proposed that IL-2 may induce the de novo expression of proteins that act between CD38 and the lytic machinery in NK cells [10]. The MAPK signalling pathway may be a potential candidate, as this pathway is activated by both IL-2 and major histocompatibility complex-1 receptor [16, 58], and may mimic mediation of the Ca^2+^-dependent steps of NK cytotoxicity. Importantly, we have previously shown a significant change in the MAPK Ca^2+^ dependent pathways for NK lysis in ME/CFS patients [45, 46].

Pharmacologically, TRPM2 currents can be inhibited by altering the production of TRPM2 secondary messengers, such as ADPR [24]. A former in vivo investigation evidenced 8-Br-ADPR as the sole secondary messenger to antagonise TRPM2 by blocking sustained tumour-induced Ca^2+^ signals and degranulation by western blot and confocal microscopy [28].

**Fig. 4:**
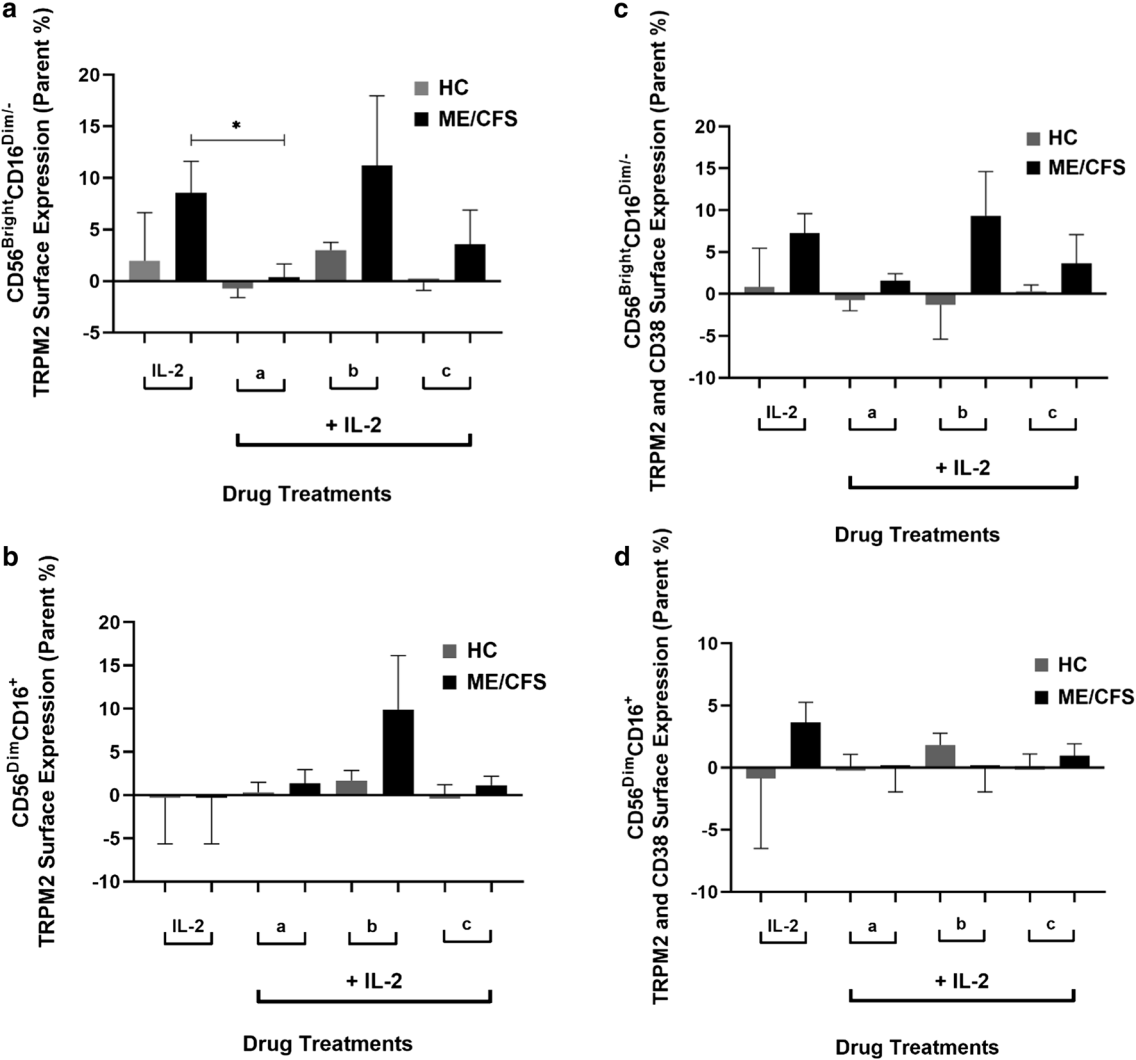
Pharmacological effect of 8-Br-ADPR and *N*_6_-Bnz-cAMP drug treatment on TRPM2 and CD38 surface expression on NK cell subsets. On CD56^Bright^CD15^Dim/−^ NK cells (**a**), TRPM2 surface expression significantly decreased following treatment with 8-Br-ADPR within the ME/CFS group. No significant difference was observed on the CD56^Dim^CD16^+^ NK cell subset for TRPM2 surface expression between and within groups (**b**). Moreover, no significant differences between or within groups were found on theCD56^Bright^CD15^Dim/−^ (**c**) and CD56^Dim^CD16^+^ (**d**) NK cell subsets for dual expression with TRPM2

Within our ME/CFS group, TRPM2 surface expression significantly decreased following 8-Br-ADPR treatment (ADPR antagonist) on CD56^Bright^CD16^Dim/−^ NK cells (Fig. 4a). Therefore, TRPM2 surface expression can be antagonised, but not effectively stimulated following agonists, such as *N*_6_-Bnz-cAMP (Fig. 4). Interestingly, this result differed with our previous TRPM3 surface expression findings following drug treatment from ME/CFS patients [45, 47]. Following 2‐aminoethoxydiphenyl borate treatment (non-selective TRPM inhibitor), TRPM3 surface expression remained unchanged on both NK cell subsets. Conversely, pregnenolone sulfate (TRPM3 agonist) treatment significantly increased TRPM3 surface expression on both NK cell subsets. Together, these findings reinforce the importance of Ca^2+^ for efficient TRP channel activity and highlight a consistent Ca^2+^ signalling dysregulation caused by impairments in TRPM ion channel expression and activity in ME/CFS patients. Moreover, as 8-Br-ADPR was administrated with IL-2, the selective diminishment of the TRPM2 ion channel may be due to the upregulation of the IL-2Rα on the CD56^Bright^CD16^Dim/−^ subset.

Comparatively, no changes in NK cell cytotoxicity were observed in either group following 8-Br-ADPR and *N*_6_-Bnz-cAMP treatment (Fig. 3). These results may reflect the limited availability of specific pharmacological drugs to modulate TRPM2 ion channels. An extensive list of pharmacological agents have been reviewed in the literature including: 8-Br-cADPR (cADPR antagonist) [29], 8-Br-ADPR (ADPR antagonist) [30], *N*_6_-Bnz-cAMP (indirect CD38 agonist), nifedipine, econazole [31], flufenamic acid [31], imidazole antifungal agents [31], anthranilic acid, N-(p-amycinnamoyl) [32] and 2-APB [32]. Unfortunately, majority of these molecules are insufficiently potent and do not exhibit high TRPM2 specificity [24], which may explain the absent changes in NK cell cytotoxicity following drug treatment in both groups (Fig. 2).

An alternative rationale may involve different TRPM2 splice variants in ME/CFS patients. Within the literature, three primary TRPM2 isoforms have been established: SSF-TRPM2, TRPM2-ΔC and TRPM2-ΔN [41, 59]. Although the full-length TRPM2 can be activated by ADPR, NAD^+^ and H_2_O_2_, TRPM2 spliced isoforms cannot be significantly stimulated by these same activators [60–62]. Interestingly, Du et al. established that 10 µM [Ca^2+^]_i_ can activate TRPM2-N, TRPM2-C, and TRPM2-N/C spliced isoforms in a concentration-dependent manner. These results suggest that [Ca^2+^]_i_ may serve as an alternative in vivo activator of both full and spliced isoforms of TRPM2, thus conferring their physiological functions [63]. Whether spliced isoforms can form functional channels remains to be determined [60–62].

Our results are considered preliminary due to our small sample size. Resultantly, our significant findings warrant further investigation with a larger cohort as a key future direction. Importantly, a significant limitation within ME/CFS research is the absence of an in vivo model and/or cell line to represent this multifactorial disorder. Consequently, current in vitro methodologies are restricted to studying isolated cells from criteria-based ME/CFS patients. One method to target specific immunological pathways from patient cells is through pharmacological modulation. However, if the mechanism of action of these drugs is unknown or non-specific, severe limitations arise in the ability to analyse accurate and reliable data. Therefore, a vital future direction is the development of pharmacotherapeutic drugs with high efficacy and specificity to TRPM2. Access to these agents will enable the use of more sophisticated applications such as whole cell electrophysiology using patch clamp techniques. Genetic methodologies are an additional future direction to understand the role of TRPM2 in Ca^2+^ signalling and NK cell function.

## Conclusion

This pilot study is the first to identify and characterise TRPM2 and CD38 surface expression on human NK cell subsets in vitro from ME/CFS patients. This investigation is also the first to examine the effects of IL-2, 8-Br-ADPR and *N*_6_-Bnz-cAMP drug treatment on TRPM2 and CD38 surface expression, as well as NK cell cytotoxicity. These new findings revealed a significant overexpression of TRPM2 on NK cells in ME/CFS patients. An overexpression in TRPM2 may function as a compensatory mechanism to alert a dysregulation in Ca^2+^ homeostasis and improve NK cell function. However, as baseline NK cell cytotoxicity was significantly reduced in ME/CFS patients compared with HCs, an impairment in the TRPM2 ion channel may result in alterations in [Ca^2+^]_i_, which is fundamental in driving NK cell cytotoxicity.

Differential expression of TRPM2 between NK cell subtypes may provide evidence for their role in the pathomechanism involving NK cell cytotoxicity activity in ME/CFS. The clinical potential of these results to develop a biological marker and drug interventions is yet to be determined.

## Supplementary information

**Additional file 1: Figure S1.** NK cells were stained with CD3^−^ APC-H7 (0.5 µg/5 µl) and CD56-Pe-Cy7 (0.25 µg/5 µl) antibodies prior to acquirement by flow cytometry. Data are represented as mean ± SEM using Mann Whittney U tests.

**Additional file 2: Figure S2.** Normalisation of TRPM2 and CD38 surface expression on NK cell subsets by flow cytometry. **(A)** Normal rabbit serum was used at comparable dilutions as the primary TRPM2 antibody (1:50) to measure TRPM2 and dual surface expression with CD38 on NK cell subsets. **(B)** Normalised TRPM2 and TRPM2/CD38 surface expression was calculated by compensating the percentage of fluorescence spill over into the B525_50 (TRPM2) and V525_50 (CD38) detectors from the TRPM2 antibody stained tube on both NK subsets.

## Data Availability

The datasets generated and/or analysed during the current study are not publicly available due to confidentiality agreements but are available from the corresponding author on reasonable request.

## Abbreviations

7-AAD: 7-amino-actinomycin
8-Br-ADPR: 8-bromoadenosine phosphoribose
ADPR: adenosine diphosphoribose
BMI: body mass index
BSA: bovine serum albumin
BD: becton dickinson
Ca^2+^: calcium
[Ca^2+^]_i_: intracellular calcium
cADPR: cyclic adenosine diphosphate ribose
cAMP: cyclic adenosine monophosphate
EDTA: ethylendiaminetetraacetic acid
FBS: fetal bovine serum
FITC: fluorescein isothiocyanate
H_2_O_2_: hydrogen peroxide
hr: hour
IL-2: interleukin-2
IL-2R: interleukin-2 receptor
JAK: janus tyrosine kinase
K^+^: potassium
LAK: lymphokine-activated killing
min: minute
*N*_6_-Bnz-cAMP: *N*_6_-benzoyladenosine-3′,5′-cyclic monophosphate
Na^2+^: sodium
NAADP: nicotinic acid adenine dinucleotide phosphate
NAD^+^: nicotinamide adenine dinucleotide
NCNED: national centre of neuroimmunology and emerging diseases
NK: natural killer
MAPK: mitogen-activated protein kinase
ME/CFS: myalgic encephalomyelitis
PBMCs: peripheral blood mononuclear cells
PI3K: phosphoinositide 3-kinase
PKA: protein kinase A
PKH-26: paul karl horan-26
RBC: red blood cell
RPMI: roswell park memorial institute medium
SF-36: short-form health survey
SOCE: store operated calcium entry
STAT: signal transducer and activator of transcription
TG: thapsigargin
TRPM2: transient receptor potential melastatin 2
WBC: white blood cell
