# Supplementary material for: Transient receptor potential melastatin 2 channels are overexpressed in myalgic encephalomyelitis/chronic fatigue syndrome patients
Source: J Transl Med. 2019 Dec 3;17:401. doi: 10.1186/s12967-019-02155-4 (PMC6891975; doi:10.1186/s12967-019-02155-4)
Supplement: Supplementary file 1 — Additional file 1: Figure S1. NK cells were stained with CD3− APC-H7 (0.5 µg/5 µl) and CD56-Pe-Cy7 (0.25 µg/5 µl) antibodies prior to acquirement by flow cytometry. Data are represented as mean ± SEM using Mann Whittney U tests. [file 12967_2019_2155_MOESM1_ESM.docx]

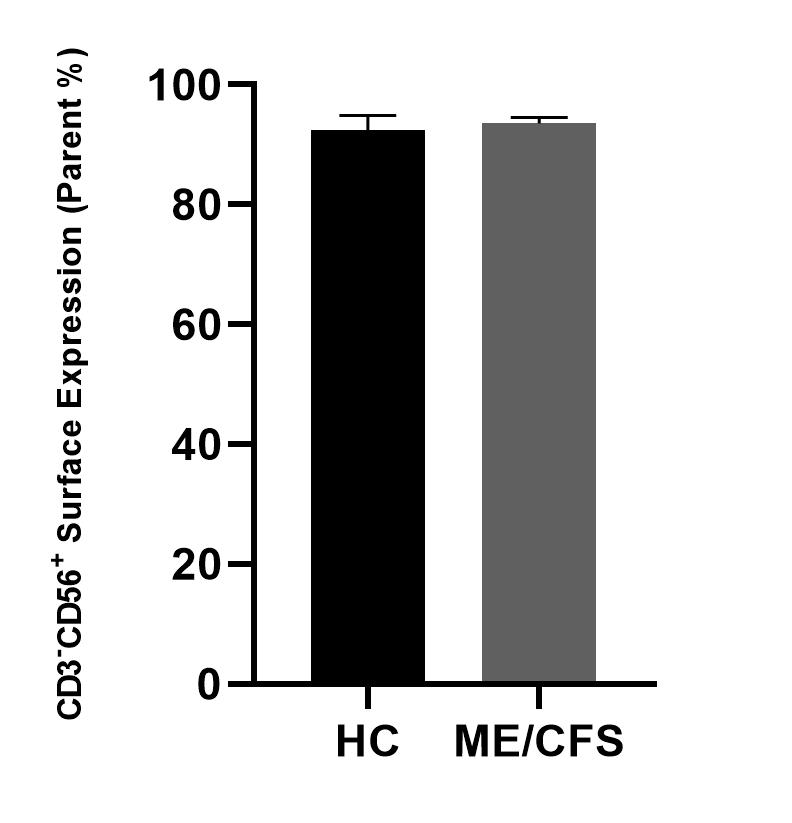


Additional file 1: Figure S1. NK cells were stained with CD3^-^APC-H7 (0.5µg/5µl) and CD56-Pe-Cy7 (0.25µg/5µl) antibodies prior to acquirement by flow cytometry. Data are represented as mean ± SEM using Mann Whittney U tests.
