# Supplementary material for: Transient receptor potential melastatin 2 channels are overexpressed in myalgic encephalomyelitis/chronic fatigue syndrome patients
Source: J Transl Med. 2019 Dec 3;17:401. doi: 10.1186/s12967-019-02155-4 (PMC6891975; doi:10.1186/s12967-019-02155-4)
Supplement: Supplementary file 2 — Additional file 2: Figure S2. Normalisation of TRPM2 and CD38 surface expression on NK cell subsets by flow cytometry. (A) Normal rabbit serum was used at comparable dilutions as the primary TRPM2 antibody (1:50) to measure TRPM2 and dual surface expression with CD38 on NK cell subsets. (B) Normalised TRPM2 and TRPM2/CD38 surface expression was calculated by compensating the percentage of fluorescence spill over into the B525_50 (TRPM2) and V525_50 (CD38) detectors from the TRPM2 antibody stained tube on both NK subsets. [file 12967_2019_2155_MOESM2_ESM.docx]

**ME/CFS PATIENT**

**HC PARTICIPANT**

1. **Normal Rabbit Serum**

1. **TRPM2 Antibody Stained**

Additional file 2: Figure S2. Normalisation of TRPM2 and CD38 surface expression on NK cell subsets by flow cytometry. **(A)** Normal rabbit serum was used at comparable dilutions as the primary TRPM2 antibody (1:50) to measure TRPM2 and dual surface expression with CD38 on NK cell subsets. **(B)** Normalised TRPM2 and TRPM2/CD38 surface expression was calculated by compensating the percentage of fluorescence spill over into the B525_50 (TRPM2) and V525_50 (CD38) detectors from the TRPM2 antibody stained tube on both NK subsets.
